# Dual Signal-Enhanced Electrochemiluminescence Strategy Based on Functionalized Biochar for Detecting Aflatoxin B1

**DOI:** 10.3390/bios13090846

**Published:** 2023-08-25

**Authors:** Lin Tian, Yuying Shi, Yanan Song, Huilin Guan, Yunxiao Li, Rui Xu

**Affiliations:** 1 Provincial Key Laboratory of Rural Energy Engineering in Yunnan, Yunnan Normal University, Kunming 650500, China; tianlin@ynnu.edu.cn; 2 School of Energy and Environment Science, Yunnan Normal University, Kunming 650500, China; shiyuying0583@126.com (Y.S.); 2223160013@ynnu.edu.cn (Y.S.); 3410@ynnu.edu.cn (H.G.); 3 Yunnan Provincial Observation and Research Station of Soil Degradation and Restoration for Cultivating Plateau Traditional Chinese Medicinal Plants, Yunnan Normal University, Kunming 650500, China

**Keywords:** biochar, MIL-88B(Fe)-NH_2_, Aflatoxin B1, electrochemiluminescence, immunosensors, food analysis

## Abstract

Metal-organic frameworks (MOFs) are often used as carriers in the preparation of electrochemiluminescent (ECL) materials, and ECL materials stabilized in the aqueous phase can be prepared by encapsulating chromophores inside MOFs by an in situ growth method. In this study, nanocomposites MIL-88B(Fe)-NH_2_@Ru(py)_3_^2+^ with excellent ECL response were prepared by encapsulating Tris(2,2′-bipyridine)ruthenium dichloride (Ru(py)_3_^2+^) inside MIL-88B(Fe)-NH_2_ using the one-step hydrothermal method. MIL-88B(Fe)-NH_2_ possesses abundant amino groups, which can accelerate the catalytic activation process of K_2_S_2_O_8_, and its abundant pores are also conducive to the enhancement of the transmission rate of co-reactant agents, ions, and electrons, which effectively improves the ECL efficiency. In order to obtain more excellent ECL signals, we prepared aminated biochar (NH_2_-biochar) using Pu-erh tea dregs as precursor and loaded gold nanoparticles (Au NPs) on its surface as substrate material for modified electrodes. Both NH_2_-biochar and Au NPs can also be used as a co-reactant promoter to catalyze the activation process of co-reactant K_2_S_2_O_8_. Therefore, a sandwich-type ECL immunosensor was prepared based on a dual signal-enhanced strategy for the highly sensitive and selective detection of aflatoxin B1 (AFB1). Under the optimal experimental conditions, the sensitive detection of AFB1 was achieved in the range of 1 pg·mL^−1^~100 ng·mL^−1^ with a detection limit of 209 fg·mL^−1^. The proposed dual signal-enhanced ECL immunosensor can provide a simple, convenient, and efficient method for the sensitive detection of AFB1 in food and agricultural products.

## 1. Introduction

Food safety is always a major livelihood issue and a priority concern in every country. Generally, food safety involves all segments of food production, processing, packaging, transportation, and storage [1,2]. Aflatoxin B1 (AFB1), as one of the most popular and toxic contaminants, may appear in every segment, especially for grain crops such as corn, peanuts, oils crops, or their processed products [3,4,5]. Thus, maximum admissible levels for AFB1 in food and feed have been set by many countries [6,7]. A variety of sensors have been applied to the sensitive detection of pollutants and biomarkers [8,9], in which an electrochemiluminescence (ECL) immunosensor method attract more and more attention in the fields of contaminant and biomarker detection, as there are many exclusive merits for the ECL immunosensor, such as simplified operation, high sensitivity, and rapid optical response [10,11,12]. Therefore, to meet the great requirements of trace, rapid and high throughout detection of AFB1, the ECL immunosensing method would be a competitive analytical assay.

In recent years, more and more research teams have developed various assays with good performance for fabricating ECL immunosensors to detect AFB1 [13,14,15,16,17]. According the published works, the detection process could be roughly divided into three categories: signal enhancement [18], signal quenching [19], and signal masking [20]. Among them, the signal enhancement type attracted more attention due to the fact that it can provide a greater signal and, thus, higher sensitivity [21]. To achieve this, not only functional materials with excellent performance but also assembly methods still have a long way to go. Up to now, much work has been done and good results have been obtained [22]. For example, the Ru(bpy)_3_^2+^ (bpy = 2,2′-bipyridine)-based ECL system offers high luminescence efficiency and chemical stability. However, the distance of electronic transmission between the Ru(bpy)_3_^2+^ and its co-reactant will directly affect the ECL signal. Recently, Li et al. reported the ECL switching of Ru(bpy)_3_^2+^ by tuning its distance from Ferrocene (Fc) allows quenching and enhancement of the ECL signal, which proves that the distance of electronic transmission between Ru(bpy)_3_^2+^ and the catalysts or the quenchers will affect the enhancement of the ECL signal [15]. Another interfering factor for the ECL signal is the stable immobilization of Ru(bpy)_3_^2+^. A recent work published by Zhang et al. reported the ECL properties of Ru(bpy)_3_^2+^ encapsulated multifunctional metal organic frameworks (Ru-MOFs) [23]. In this study, Ru(bpy)_3_^2+^ was encapsulated in internal pores by in situ-grown MOFs, allowing Ru(bpy)_3_^2+^ to produce a stable ECL response in an aqueous solution. Therefore, both preparing substrate materials with superior catalytic property for catalyzing co-reactant and larger specific surface area for immobilizing antibodies and preparing labelling materials with excellent ECL property are highly demanded for the development of ECL immunosensors.

To get great ECL signals, we focused our research on metal organic framework (MOF) materials since it might provide a large specific surface area for stably loading large amounts of luminophores and good catalytic actives for catalyzing the co-reactant in order to greatly improve the ECL signal [24]. MOFs have been gradually introduced in the fabrication of ECL (bio)sensors in recent years, although they are already popular in the research fields of catalysts and energy. Their merits for enhancing ECL signals are being discovered and attracting more and more focus. As a recent good example, Yuan and Zhuo et al. prepared an isoreticular metal organic framework-3 (IRMOF-3) with 2-amino terephthalic acid (NH_2_-BDC) for fabricating a signal enhancing ECL immunosensor [25]. In their work, IRMOF-3 was used for loading a large amount of CdTe via the encapsulating effect and internal/external decoration. Furthermore, it was used as a novel co-reactant accelerator, which could promote the conversion of co-reactant S_2_O_8_^2–^ to SO_4_^•−^ (i.e., sulfate radical anion), thus boosting the ECL emission of CdTe quantum dots (QDs).

Furthermore, based on our previous investigation and summarization [26], we found that porous activated biochar with the merits of good chemical stability and large surface area was appropriately chosen as the support material [27]. Essentially, the biochar obtained from renewable biomass through pyrolysis or carbonization processes under a low-concentration oxygen atmosphere is porous, eco-friendly, and low-cost [28,29]. Especially, after the biochar is chemically activated, the activated biochar (AB) shows superior properties of a highly functionalized surface, promoting high adsorptivity and interaction capacity [30]. Inspired by this, we proposed using AB obtained from the biomass of Pu-erh tea produced in the Yunnan Province in China as the platform for the development of ECL immunosensor.

**Scheme 1:**
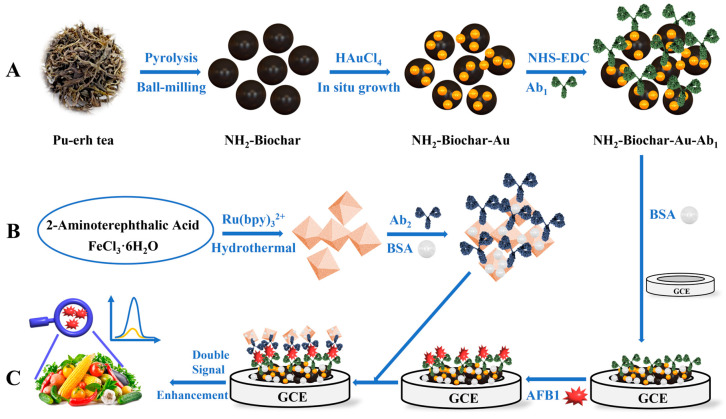
Fabrication process of the ECL immunosensor: (**A**) the preparation of NH_2_-Biochar-Au-Ab_1_, (**B**) the preparation of MIL-88B(Fe)-NH_2_@Ru(bpy)_3_^2+^-Ab_2_-BSA, and (**C**) the detection of AFB1.

From the above analysis, a signal enhancement type of ECL immunosensor for the detection of AFB1 was developed in this work. As shown in Scheme 1, firstly, ammonia activated biochar particles (NH_2_-Biochar) were prepared from Pu-erh tea biomass by low temperature pyrolysis and successively grounded via a ball-milling process. To improve its conductivity and loading capacity, Au nanoparticles (Au NPs) were in-situ grown on it to obtain the compound material NH_2_-Biochar (NH_2_-Biochar-Au). Following that, the AFB1 primary antibodies (Ab_1_) was loaded on the NH_2_-Biochar-Au via NHS-EDC connection (NH_2_-Biochar-Au-Ab_1_). Secondly, due to the ammonia iron-based metal-organic framework (MIL-88B(Fe)-NH_2_), nanoparticles can be easily synthesized with controlled size and peroxidase-like catalytic activity [31], and MIL-88B(Fe)-NH_2_ used as the co-reactant promoter and carrier to load Ru(bpy)_3_^2+^ were prepared and named MIL-88B(Fe)-NH_2_@Ru(bpy)_3_^2+^. Following that, the MIL-88B(Fe)-NH_2_@Ru(bpy)_3_^2+^ was used as the signal probe to label AFB1 secondary antibodies (Ab_2_). Lastly, the sandwich type of ECL immunosensor was fabricated and investigated.

## 2. Results and Discussion

### 2.1. Characterization of MIL-88B(Fe)-NH_2_@Ru(bpy)_3_^2+^ and NH_2_-Biochar-Au

**Figure 1:**
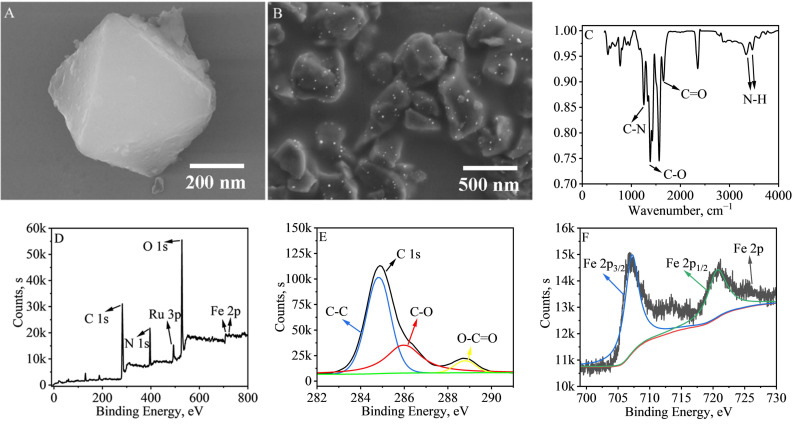
SEM images of (**A**) MIL-88B(Fe)-NH_2_@Ru(bpy)_3_^2+^, and (**B**) NH_2_-Biochar-Au. (**C**) FT-IR spectrum of MIL-88B(Fe)-NH_2_@Ru(bpy)_3_^2+^. (**D**) XPS spectra of MIL-88B(Fe)-NH_2_@Ru(bpy)_3_^2+^, (**E**) C 1s and (**F**) Fe 2p.

Figure 1A shows the SEM characterization image of MIL-88B(Fe)-NH_2_@Ru(bpy)_3_^2+^, which is a classical octahedral structure with a particle size of about 400 nm. The structure is not significantly changed compared with that of MIL-88B(Fe)-NH_2_, indicating that the encapsulated Ru(bpy)_3_^2+^ in MIL-88B(Fe)-NH_2_ was successfully prepared [32]. To investigate the functional group species of MIL-88B(Fe)-NH_2_@Ru(bpy)_3_^2+^, we characterized them using the FT-IR spectroscopy method, and the results are shown in Figure 1C. The two characteristic peaks near 3300–3500 cm^−1^ are generated by the primary amine N-H stretching vibration, and the characteristic peak at 1154 cm^−1^ is generated by the C-N stretching vibration, indicating that MIL-88B(Fe)-NH_2_ possesses an amino group. The characteristic peaks of C-O at 1382 cm^−1^ and C=O at 1652 cm^−1^ indicate that MIL-88B(Fe)-NH_2_ also possesses a carboxyl group, suggesting that MIL-88B(Fe)-NH_2_ can be attached to the antibody through an amide bond [33]. As shown in Figure 1D–F, the XPS profiles of MIL-88B(Fe)-NH_2_@Ru(bpy)_3_^2+^, the C 1s profile and the Fe 2p profile are shown, respectively. As shown in Figure 1D, the presence of C, N, Ru, O, and Fe, and the characteristic peak of Ru 3p near 484 eV all indicate the successful preparation of MIL-88B(Fe)-NH_2_@Ru(bpy)_3_^2+^ [19]. The characteristic peaks at 709.7 and 723.5 eV are two orbital peaks of Fe 2p, indicating that the elemental iron in MIL-88B(Fe)-NH_2_@Ru(bpy)_3_^2+^ is in the form of Fe^3+^. The characteristic peaks at 284.8, 287.3 and 288.9 eV are those of C-C, C-O and O-C=O, respectively, indicating the presence of carboxyl groups in MIL-88B(Fe)-NH_2_@Ru(bpy)_3_^2+^. The result is consistent with the information in the FT-IR spectrum [34,35]. NH_2_-Biochar-Au in this work was prepared using a general in situ growth process. As shown in Figure S1 and Figure 1B, Au NPs with uniform size are well composited onto the NH_2_-Biochar.

### 2.2. Mechanism of the ECL Immunosensor

**Figure 2:**
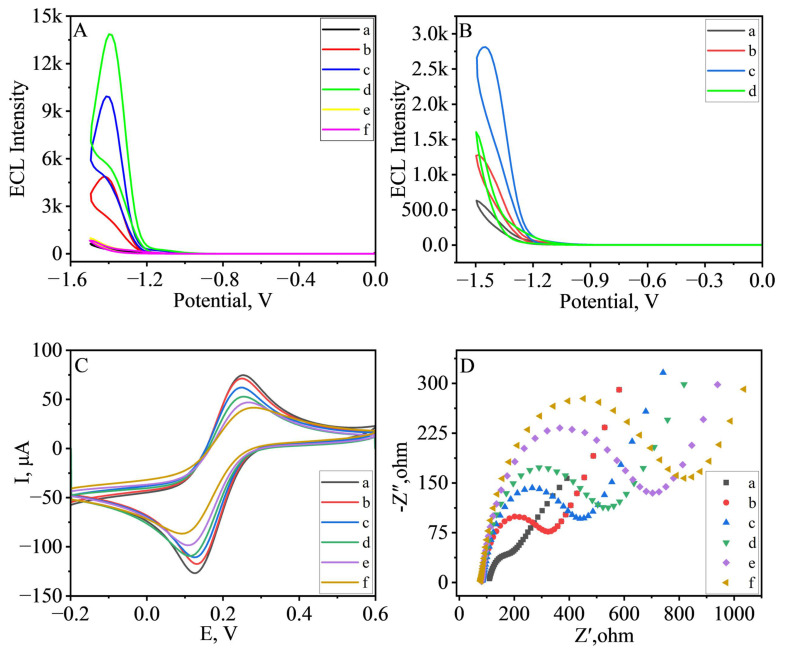
(**A**) ECL responses of (a) GCE, (b) GCE/MIL-88B(Fe)-NH_2_@Ru(bpy)_3_^2+^, (c) GCE/NH_2_-Biochar/MIL-88B(Fe)-NH_2_@Ru(bpy)_3_^2+^, and (d) GCE/NH_2_-Biochar-Au/MIL-88B(Fe)-NH_2_@Ru(bpy)_3_^2+^ (in PBS containing 90 mmol·L^−1^ K_2_S_2_O_8_). ECL responses of (e) GCE/MIL-88B(Fe)-NH_2_, (f) GCE/NH_2_-Biochar-Au (in PBS). (**B**) ECL responses of (a) GCE, (b) GCE/NH_2_-Biochar, (c) GCE/NH_2_-Biochar-Au, and (d) GCE/MIL-88B(Fe)-NH_2_ (in PBS containing 90 mmol·L^−1^ K_2_S_2_O_8_). (**C**) Cyclic Voltammetry Results (in 5 mM Fe(CN)_6_^3−^ and 0.1 M KNO_3_) and (**D**) Electrochemical Impedance Spectroscopy Results (in 2.5 mM Fe(CN)_6_^3−/4−^ and 0.1 M KCl) of (a) bare GCE, (b) GCE/NH_2_-Biochar-Au, (c) GCE/NH_2_-Biochar-Au/Ab_1_, (d) GCE/NH_2_-Biochar-Au/Ab_1_/BSA, (e) GCE/NH_2_-Biochar-Au/Ab_1_/BSA/AFB1, and (f) GCE/NH_2_-Biochar-Au/Ab_1_/BSA/AFB1/MIL-88B(Fe)-NH_2_@Ru(bpy)_3_^2+^-Ab_2_-BSA. The concentration of AFB1 used for the test was 50 ng·mL^−1^.

Curves a, b, c, and d in Figure 2A show the ECL response curves of GCE, MIL-88B(Fe)-NH_2_@Ru(bpy)_3_^2+^, NH_2_-Biochar/MIL-88B(Fe)-NH_2_@Ru(bpy)_3_^2+^, and NH_2_-Biochar-Au/MIL-88B(Fe)-NH_2_@Ru(bpy)_3_^2+^ in PBS containing 90 mmol·L^−1^ K_2_S_2_O_8_. In the figure we can find almost no ECL response in GCE (curve a). When only MIL-88B(Fe)-NH_2_@Ru(bpy)_3_^2+^ was coated on the surface of GCE, there was a smaller ECL response (curve b). When NH_2_-Biochar/MIL-88B(Fe)-NH_2_@Ru(bpy)_3_^2+^ was coated on the surface of GCE, the ECL response (curve c) doubled in size compared to when only MIL-88B(Fe)-NH_2_@Ru(bpy)_3_^2+^ was coated. The ECL response obtained when coated with NH_2_-Biochar-Au/MIL-88B(Fe)-NH_2_@Ru(bpy)_3_^2+^ was again enhanced. The synergistic catalytic effect of NH_2_-Biochar and Au NPs resulted in the final obtained ECL response (curve d) being about 2.6 times higher than that obtained when coated with MIL-88B(Fe)-NH_2_@Ru(bpy)_3_^2+^. Curves e, f in Figure 2A show the ECL response curves of MIL-88B(Fe)-NH_2_ and NH_2_-Biochar-Au in PBS, respectively. We can see that MIL-88B(Fe)-NH_2_ and NH_2_-Biochar-Au have almost no ECL response in PBS. Therefore, it can be inferred that the signal enhancement effect is due to the catalytic activation of K_2_S_2_O_8_ by MIL-88B(Fe)-NH_2_ and NH_2_-Biochar-Au.

To investigate the catalytic ability of NH_2_-Biochar, NH_2_-Biochar-Au, and MIL-88B(Fe)-NH_2_ on the co-reactant K_2_S_2_O_8_, we obtained their ECL responses by simultaneous CV scanning. The results are shown in Figure 2B, where the bare electrode has only a low ECL response (Figure 2B, curve a), and when NH_2_-Biochar is coated on the electrode surface, the ECL response is enhanced by a factor of two compared to the GCE (Figure 2B, curve b). The ECL response was about 4.5 times higher than that of GCE when NH_2_-Biochar@Au was coated on the electrode surface (Figure 2B, curve c). Not only that, when MIL-88B(Fe)-NH_2_ was coated on the electrode surface, the ECL response was about 2.7 times higher than that of GCE (Figure 2B, curve d). The above results indicate that MIL-88B(Fe)-NH_2_ can promote the reduction of S_2_O_8_^2−^ in the NH_2_-Biochar-Au/MIL-88B(Fe)-NH_2_@Ru(bpy)_3_^2+^ system. Likewise, NH_2_-Biochar-Au showed the same effect. In conclusion, NH_2_-Biochar-Au and MIL-88B(Fe)-NH_2_ can be used as co-reactant promoters to further enhance the ECL reaction of Ru(bpy)_3_^2+^ when S_2_O_8_^2−^ is used as co-reactant. The ECL reaction mechanism of MIL-88B(Fe)-NH_2_@Ru(bpy)_3_^2+^ can be summarized as follows [36,37,38]:

Ru(bpy)_3_^2+^ + e^−^ → Ru(bpy)_3_^+^

S_2_O_8_^2−^ + e^−^ → SO_4_^2−^ + SO_4_^•−^

Ru(bpy)_3_^+^ + SO_4_^•−^ → Ru(bpy)_3_^2+*^ + SO_4_^2−^

Ru(bpy)_3_^2+*^ → Ru(bpy)_3_^2+^ + *hv*

### 2.3. Characterization of Layer Modification Process of ECL Immunosensor

To verify the successful construction of the ECL immunosensor, the techniques of Cyclic Voltammetry (CV) and Electrochemical Impedance Spectroscopy (EIS) were used to characterize the layer modification process in the construction of the immunosensor, and the results are shown in Figure 2C,D. It can be observed from Figure 2C that the bare GCE has the highest peak current. As NH_2_-Biochar-Au-Ab_1_, BSA, AFB1, and MIL-88B(Fe)-NH_2_@Ru(bpy)_3_^2+^-Ab_2_-BSA, which contain macromolecular proteins, are modified to the electrode surface, the peak current decreases continuously. Similarly in Figure 2D, as the electrode is modified layer by layer, its electron transfer resistance increases. This is because AFB1, Ab_1_, Ab_2_, and BSA are all large molecules with poor electrical conductivity, and the results indicate that the immunosensor was successfully constructed.

### 2.4. Selection of Measurement Condition

Experimental condition is an important factor affecting the excellent performance of ECL immunosensors in use, and the best performance can be obtained by testing under optimal experiment conditions. A controlled variable approach was used to select optimal parameters for the immunosensor during the detection.

**Figure 3:**
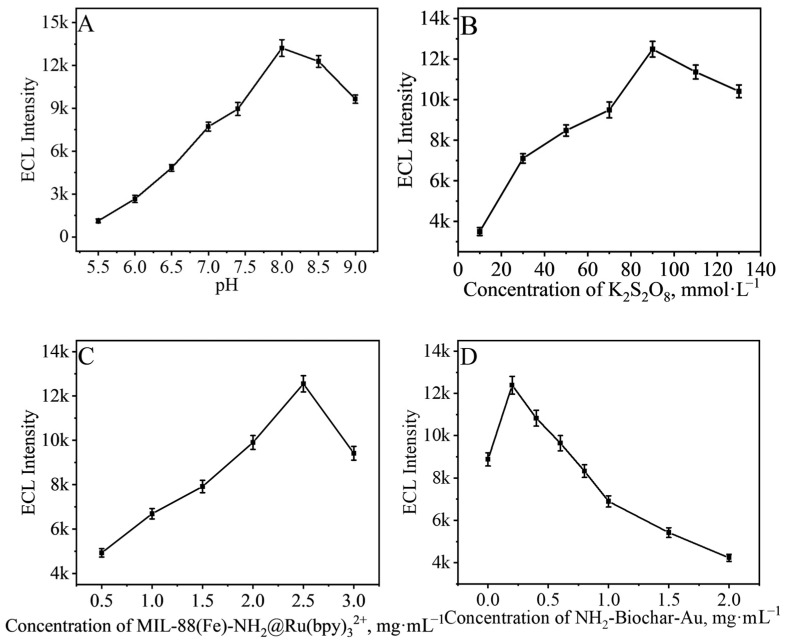
Effects of (**A**) pH, (**B**) concentration of K_2_S_2_O_8_, (**C**) concentration of MIL-88B(Fe)-NH_2_@Ru(bpy)_3_^2+^, and (**D**) concentration of NH_2_-Biochar-Au on the ECL response.

The value of pH is an important factor affecting the signal of the immunosensor, and we first selected the pH of the substrate and tested the performance of the immunosensor in PBS containing 90 mmol·L^−1^ K_2_S_2_O_8_ at different pH. The results are shown in Figure 3A. The ECL signal of the immunosensor increased as the pH value increased, and the best ECL signal was obtained when pH = 8.0, and the signal gradually decreased when the pH value continually increased, so we chose pH = 8.0 of PBS for the subsequent tests.

In this study, K_2_S_2_O_8_ was used as a co-reactant, and an excess of K_2_S_2_O_8_ would inhibit its own activation process. Therefore, the performance of the immunosensor in PBS containing different concentrations of K_2_S_2_O_8_ at pH = 8.0 was tested. The results are shown in Figure 3B, where the immunosensor obtained the best ECL signal at the K_2_S_2_O_8_ concentration of 90 mmol·L^−1^.

Moreover, the concentrations of MIL-88B(Fe)-NH_2_@Ru(bpy)_3_^2+^ and NH_2_-Biochar-Au for the construction of the immunosensor were also selected. As shown in Figure 3C, when the concentration of MIL-88B(Fe)-NH_2_@Ru(bpy)_3_^2+^ was at 2.5 mg·mL^−1^, the best ECL signal was obtained by the immunosensor. However, the signal decreased with continued increase of the concentration of MIL-88B(Fe)-NH_2_@Ru(bpy)_3_^2+^. This might be because of the masking of self-luminescence caused by excess MIL-88B(Fe)-NH_2_@Ru(bpy)_3_^2+^. The concentration of NH_2_-Biochar-Au reached the best ECL signal at 0.2 mg·mL^−1^, and, thus, 0.2 mg·mL^−1^ of NH_2_-Biochar-Au was selected for the construction of the immunosensor.

### 2.5. Performance of the ECL Immunosensor

**Figure 4:**
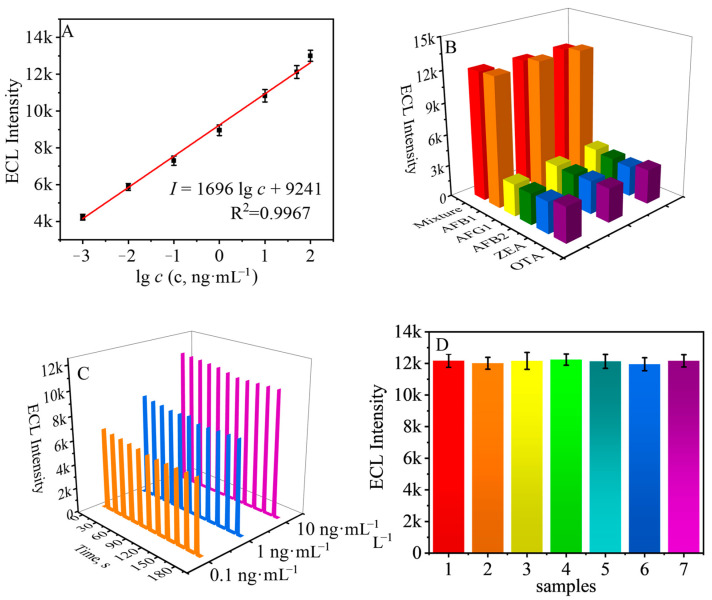
(**A**) Working curves of the designed ECL immunosensor in the linear range (0.001, 0.01, 0.1, 1, 10, 50, and 100 ng·mL^−1^). (**B**) Selectivity of the designed ECL immunosensor in the detection of AFB1 (50 ng·mL^−1^ for AFB1 and other interferents). (**C**) Stability of the designed ECL immunosensor in detecting different concentrations of AFB1. (**D**) Reproducibility of the designed ECL immunosensor. Error bars = SD (n = 3).

To verify the performance of the designed immunosensor, the analytical performance of the immunosensor was firstly investigated under the above optimal experimental conditions. The results are shown in Figure 4A. The obtained ECL signal was positively correlated with the logarithm of AFB1 concentration and was able to achieve sensitive detection of AFB1 in the range of 1 pg·mL^−1^~100 ng·mL^−1^. The regression equation of the obtained fitted curve was *I* = 9241 + 1696 log *c*, where *I* is the ECL intensity, *c* is the concentration of AFB1, and R^2^ = 0.9967. We also prepared a batch of signal probes without AFB1, and the detection limit of the immunosensor was calculated to be 209 fg·mL^−1^ after testing.

### 2.6. Other Parameters of the ECL Immunosensor

As shown in Figure 4B, when the detection objects were Ochratoxin A (OTA), Zearalenone (ZEA), Aflatoxin B2 (AFB2), and Aflatoxin G1 (AFG1), the ECL signals were all much smaller than that of detecting AFB1 and the mixture, indicating that the prepared ECL immunosensor has good selectivity and can accurately detect AFB1 in complicate samples.

The stability of the designed immunosensors was evaluated by testing different concentrations of AFB1. Specifically, three batches were constructed using AFB1 at concentrations of 0.1, 1, and 10 ng·mL^−1^. The results are shown in Figure 4C, and the constructed immunosensors showed stable ECL signals. Additionally, we also prepared seven batches of immunosensors using 50 ng·mL^−1^ of AFB1 under the same experimental conditions, and as the results show in Figure 4D, the prepared seven batches of immunosensors showed good reproducibility with an RSD value of only 2.71%.

In order to verify the performance of our constructed ECL immunosensor in detecting real samples, we conducted experiments using the standard addition method [39]. Firstly, the corn grains were mixed with deionized water and broken with a wall breaker, and the filtrate was obtained by filtration using a 0.22 μm syringe filter membrane, and the filtrate and deionized water were prepared into a dilute solution at a ratio of 1:100. Six sets of mixed solutions were obtained by adding 1 mL of 0.1, 0.5, 1, 2, 5, and 10 ng/mL AFB1 to 1 mL dilute solution and mixing thoroughly. Seven ECL sensors were fabricated and tested under the same experimental conditions using each group of mixed solutions, and the obtained ECL signals were combined with the working curve to calculate the concentrations. The results are shown in Table S1. The relative standard deviation (RSD) was less than 3.8% and the recoveries were between 96.0 and 106%, indicating that our prepared ECL immunosensors can be used for the detection of real samples. As shown in Table S2, we compared the constructed ECL immunosensor with other previously reported methods for the detection of AFB1, and the results showed that the ECL immunoassay prepared in this study has excellent performance in detecting AFB1.

## 3. Materials and Methods

### 3.1. Preparation of NH_2_-Biochar

The NH_2_-Biochar materials were obtained by a two-step process using the Pu-erh tea produced in Yunnan Province in China as the carbon precursor. Typically, Pu-erh tea was first washed with deionized water to remove unwanted impurities. After being dried at 60 °C, the precursors were put into a tubular furnace and carbonized at 800 °C under an N_2_ gas for 2 h (heating rate 5 °C·min^−1^). Following that, the black products were cooled down to room temperature and ground up in a Guge AD-G858 blender just as the previous work [40]. The black products were sieved to biochar powders with the diameter between 100 and 250 μm range. The prepared biochar was then polished into particles of uniform particle size using a ball milling technique [41,42,43]. Specifically, the biochar and agate balls (φ = 6 mm) were mixed well in a 500 mL agate container with a mass ratio of 3:200 and then placed in the agate container, and polished on a semi-circular planetary ball mill at 300 rpm for 5 h after adding an appropriate amount of ammonia. After the mixture was washed with DI water followed by isopropanol until neutrality, the NH_2_-Biochar was obtained through a process of successive oven drying.

### 3.2. Preparation of MIL-88B(Fe)-NH_2_@Ru(bpy)_3_^2+^

MIL-88B(Fe)-NH_2_@Ru(bpy)_3_^2+^ was prepared according to a previous study with slight modifications. NH_2_-BDC (0.25 g) and FeCl_3_·6H_2_O (0.38 g) were first added to 30 mL DMF and sonicated to dissolve them well. Then, Ru(bpy)_3_^2+^ (50 mg) was added to the mixed solution with stirring for five minutes and transferred to a 50 mL autoclave containing Teflon liner and was heated at 120 °C for 24 h. The product was collected by centrifugation and washed with DMF and ethanol alternately, and was finally dried at 60 °C overnight to obtain MIL-88B(Fe)-NH_2_@Ru(bpy)_3_^2+^.

### 3.3. Preparation of NH_2_-Biochar-Au, NH_2_-Biochar-Au-Ab_1_, and the Label of MIL-88B(Fe)-NH_2_@Ru(bpy)_3_^2+^-Ab_2_-BSA

The preparation processes are as shown in Scheme 1A,B. The specific experimental steps are provided in the Supporting Information.

### 3.4. Preparation Process of ECL Immunosensor

The preparation process of the ECL immunosensor for the detection of AFB1 is as shown in Scheme 1C. Specifically, at first, 6 μL of NH_2_-Biochar-Au-Ab_1_ was dropped onto the GCE and stored at 4 °C until slightly wetted. Then, 6 μL of 1% BSA solution was dropped on the surface of the NH_2_-Biochar-Au-Ab_1_ modified electrode and incubated at 4 °C for 2 h to block the non-specific active site on the surface of the modified electrode. The excess BSA on the electrode surface was washed off by rinsing the electrode in PBS (pH = 7.4). To enable the AFB1 to immune attach on the surface of the above electrode, 6 μL different concentrations of AFB1 were dropped onto the uppermost layer of the electrode and it was incubated for 2 h at 4 °C. Following that, the unfixed AFB1 was washed away with PBS (pH = 7.4). Finally, 10 μL 2.5 mg·mL^−1^ MIL-88B(Fe)-NH_2_@Ru(bpy)_3_^2+^-Ab_2_-BSA was dropped onto the topmost layer of the above-modified electrode to specifically bind to the AFB1 and the electrode was washed by means of PBS to completely eliminate the unattached bioconjugate.

## 4. Conclusions

In summary, we successfully prepared MIL-88B(Fe)-NH_2_@Ru(bpy)_3_^2+^ as ECL signal probe using a one-step hydrothermal method. The amino-rich MIL-88B(Fe)-NH_2_ cannot only be used as carrier to load Ru(bpy)_3_^2+^, but also as a co-reactor promoter to catalyze the activation process of the K_2_S_2_O_8_. MIL-88B(Fe)-NH_2_ possesses abundant pores that can enrich the SO4^•−^ produced by the catalytic activation of K_2_S_2_O_8_, which shortens the material transfer distance and energy transfer distance between Ru(bpy)_3_^2+^ and SO4^•−^, and effectively improves the cathodoluminescence efficiency of MIL-88B(Fe)-NH_2_@Ru(bpy)_3_^2+^. In addition, we used Pu-erh tea dregs as precursor to obtain biochar by calcination in a high-temperature and oxygen-free environment. The obtained biochar was ball-milled and aminated to prepare NH2-Biochar, and Au NPs were grown in situ on its surface to prepare NH_2_-Biochar-Au used as substrate material. The Au NPs could not only improve the conductivity of NH2-Biochar, but also provide active sites for antibody immobilization. Not only that, both NH_2_-Biochar and Au NPs can be used as co-reactant promoters to accelerate the catalytic activation process of K_2_S_2_O_8_. Through the co-catalysis between MIL-88B(Fe)-NH_2_ and NH_2_-Biochar-Au, our ECL immunosensor prepared based on dual signal-enhanced strategy was capable of efficient and sensitive detection of AFB1 in the range of 1 pg·mL^−1^~100 ng·mL^−1^, with a detection limit of 209 fg·mL^−1^. It provides a simple and efficient method for the rapid and sensitive detection of AFB1 in food and agricultural products. Compared with other methods for the detection of AFB1, our ECL immunosensor constructed based on the dual signal amplification strategy has a lower detection limit, but the construction process is more cumbersome requiring multiple washes. Therefore, we hope to develop more convenient wash-free sensors for the sensitive detection of AFB1.

## Data Availability

Not applicable.

## Supplementary Materials

The following supporting information can be downloaded at: https://www.mdpi.com/article/10.3390/bios13090846/s1, Materials and reagents; Apparatus; Measurement Procedure; Preparation Procedure; Table S1: Detection results of AFB1-spiked corn samples; Table S2: Comparison of our work and other methods; Figure S1: Characterization of Au NPs. References [44,45,46,47,48,49,50] are cited in the supplementary materials.
