# Supplementary material for: Dual Signal-Enhanced Electrochemiluminescence Strategy Based on Functionalized Biochar for Detecting Aflatoxin B1
Source: Biosensors (Basel). 2023 Aug 25;13(9):846. doi: 10.3390/bios13090846 (PMC10526187; doi:10.3390/bios13090846)
Supplement: Supplementary file 1 [file biosensors-13-00846-s001.zip › biosensors-2486128-supplementary.pdf]

biosensors
MDPI

Supplementary

Dual Signal-Enhanced Electrochemiluminescence Strategy Based on Functionalized Biochar for Detecting Aflatoxin B1

Lin Tian, Yuying Shi, Yanan Song, Huilin Guan, Yunxiao Li and Rui Xu

1. Materials and reagents

Ferric chloride hexahydrate ($FeCl_3 \cdot 6H_2O$), 1-(3-(dimethylamino) propyl)-3-ethyl carbodiimide hydrochloride (EDC), dibasic Sodium Phosphate (Na_2HPO_4), potassium dihydrogen phosphate (KH_2PO_4), potassium chloride (KCl), potassium peroxydisulfate ($K_2S_2O_8$), Chloroauric acid ($HAuCl_4$), polyvinylpyrrolidone (PVP), and Trisodium citrate ($Na_3C_6H_5O_7 \cdot 2H_2O$) were obtained from Macklin Biochemical Technology Co., Ltd. (Shanghai, China). Bovine serum albumin (BSA, >98.0%) was obtained from Sigma-Aldrich Co., Ltd. (Shanghai, China). Potassium ferricyanide ($K_3FeC_6N_6$), potassium ferrocyanide trihydrate ($K_4FeC_6N_6 \cdot 3H_2O$), N, N-dimethylformamide (DMF), Sodium borohydride ($NaBH_4$), and Aqueous ammonia (NH_4OH) were purchased from Nanjing Chemical Reagent Co., Ltd. (Nanjing, China). 2-Aminoterephthalic Acid (NH_2-BDC) and N-hydroxysuccinimide (NHS) were purchased from Aladdin Biotech Co., Ltd. (Shanghai, China). Both of the antigen-AFB1, AFB1 primary antibodies (Ab_1) and AFB1 secondary antibodies (Ab_2), were obtained from Chundubio (Wuhan, China). 0.1 M phosphate buffer saline (PBS) was prepared by mixing the stock solutions of Na_2HPO_4 and KH_2PO_4 in appropriate ratios. Ultrapure water obtained from Millipore water purification system ($\geq$ 18 MΩ cm^{-1}, Milli-Q, Millipore) was used in all assays. All chemicals were of analytical grade.

2. Apparatus

Scanning electron microscope (SEM) images and energy dispersive spectroscopy (EDS) spectrograms were obtained using a field emission SEM (Zeiss, Germany). High resolution transmission electron microscope (HRTEM) images were obtained from a JEM microscope (Hitachi, Japan). Fourier transform infrared spectroscopy (FT-IR) spectrogram was recorded by VERTEX70 Spectrometer (Bruker Co. Ger). X-ray powder diffraction (XRD) patterns were obtained by D8 advance X-ray diffractometer (Bruker AXS, Germany). Elemental composition of nanomaterials was obtained by X-ray photoelectron spectroscopy (XPS, Axis Supra, Japan). UV-vis absorbance spectra were examined with a Lambda 25 UV-vis Spectrophotometer (PerkinElmer, American). ECL signals were monitored by the MPI-E Electrochemiluminescence Analyzer (Xi'an remax Electronic Science Tech. Co. Ltd., China).

3. CV Measurement Procedure

Electrochemical measurements were performed with CHI760D electrochemical workstation (Chenhua Instrument Shanghai Co., Ltd., China) from -0.2 V to 0.6 V. During the detection process, a conventional three-electrode system was employed with a saturated calomel electrode (saturated KCl solution) as reference electrode, a platinum wire as auxiliary electrode, and a modified glass carbon electrode (GCE, Ø = 4 mm) as working electrode. The scan rate was 0.1 V/s in the detection process.

4. ECL Measurement Procedure

Before measuring, the double enhanced ECL sensing platform was incubated with different concentrations of AFB1 for 2 h. The ECL behavior was implemented by an MPI-F flow-injection chemiluminescence detector with a continuous cyclic potential scanning

from -1.5 to 0 V in 0.1mol·L^{-1} PBS (pH=7.4) containing 90 mmol·L^{-1} $K_2S_2O_8$. The photomultiplier tube was set at 600 V and the scan rate was 0.15 V/s in the detection process.

5. Synthesis of NH_2-Biochar-Au Nanocomposites

First, 50 mg prepared NH_2-Biochar was dispersed in 10 mL deionized water and made to disperse uniformly by ultrasonication; then, 7 mg PVP and 1 mL 1% $HAuCl_4$ solution were added and stirred magnetically for 5 min. Then, 80mg $Na_3C_6H_5O_7·2H_2O$ was dissolved in 5 mL deionized water, 1mg $NaBH_4$ was dissolved in 5 mL deionized water. While stirring magnetically, $Na_3C_6H_5O_7$ solution was added drop by drop to the mixed solution, and then $NaBH_4$ solution was added drop by drop to the above solution until the color of the solution did not change. Finally, the above solution was stirred overnight under dark conditions, and the product was collected by centrifugation and washed several times with deionized water and dried under vacuum at 60°C for 12 hours.

6. Preparation of NH_2-Biochar-Au-Ab_1 Bioconjugate

First, 0.2 mg prepared NH_2-Biochar-Au was dispersed in 900 μL PBS (pH=7.4), then 50 μL 400 mmol·L^{-1} EDC solution and 50 μL 100 mmol·L^{-1} NHS solution were added to the above solution and shaken for 3 h at 4°C. After centrifugation, in order to remove excess crosslinker, 950 μL PBS (pH=7.4) and 50 μL 10 μg·mL^{-1} Ab_1 solution were added and shaken overnight at 4°C. The synthesized NH_2-Biochar-Au-Ab_1 bioconjugate was collected by centrifugation and washed with PBS (pH=7.4), dispersed in 1 mL PBS (pH=7.4) and stored at 4°C for use in the next step.

7. Preparation of MIL-88B(Fe)-NH_2@$Ru(bpy)_3^{2+}$-Ab_2-BSA

2.5 mg MIL-88B(Fe)-NH_2@$Ru(bpy)_3^{2+}$ nanocomposites were added to 850 μL PBS (pH=7.4) and homogeneously dispersed by sonication, and then 50 μL EDC solution (400 mmol·L^{-1}) and 50 μL NHS solution (100 mmol·L^{-1}) were added to the mixed solution, followed by shaking at 4° C for 3 h. After removing excess crosslinker by centrifugation, 950 μL of PBS (pH=7.4) and 50 μL of 10 μg·mL^{-1} Ab_2 solution were added and shaken overnight at 4°C. After centrifugation, again to remove unreacted material, the synthesized MIL-88B(Fe)-NH_2@$Ru(bpy)_3^{2+}$-Ab_2 bioconjugate was washed with PBS (pH=7.4). Then, 950 μL PBS (pH=7.4) and 50 μL 1% BSA solution were added and shaken for 6 h at 4°C. The synthesized MIL-88B(Fe)-NH_2@$Ru(bpy)_3^{2+}$-Ab_2-BSA bioconjugate was collected by centrifugation and washed with PBS (pH=7.4), dispersed in 1 mL PBS (pH=7.4) and stored at 4°C for use in the next step.

Table S1. Detection results of AFB1-spiked corn samples.

Sample (ng/mL)	Addition (ng/mL)	Average (ng/mL, n=7)	RSD (%)	Recovery (%)
Not detected	0.1	0.098	2.1	98.0
	0.5	0.53	2.7	106.0
	1	0.96	3.6	96.0
	2	2.08	2.4	104.0
	5	4.88	2.9	97.6
	10	10.22	3.8	102.2

Table S2. Comparison of our work and other methods.

Methods	Linear range (ng/mL)	Detection limit (pg/mL)	Ref.
DPV	0.01 to 20	8	[1]
PEC	0.005 to 15	1.7	[2]
PEC	0.01 to 1000	2.95	[3]
ECL	0.01 to 100	2.63	[4]
ECL	0.005 to 10	5	[5]
FL	0.001 to 0.05	3	[6]
FL	0 to 180	350	[7]
Colorimetric	1 to 6	180	[8]
ECL	0.001 to 100	0.209	This work

DPV: differential pulse voltammetry
PEC: photoelectrochemical
FL: fluorescence

8. TEM images and particle size distribution statistics of Au NPs

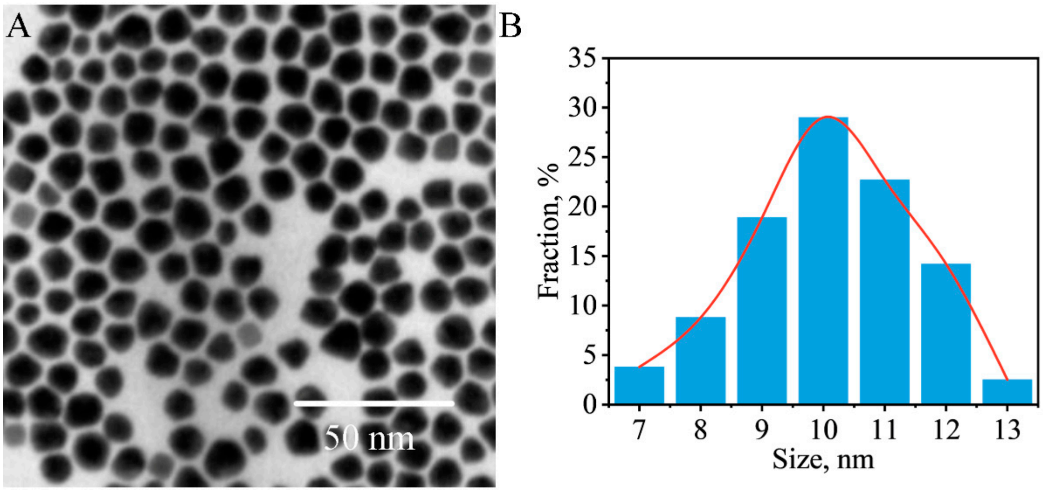

Figure S1. (A) TEM image of Au NPs. (B) Statistical graph of particle size distribution of Au NPs.

References

1. Ou, G.; Zhao, A.; Liao, H.; Zhang, Z.; Xiao, F. Au nanopartics decorated urchin-like Bi2S3 on graphene wrapped carbon fiber microelectrode: Towards electrochemical immunosensor for sensitive determination of aflatoxin B1. *J. Electroanal. Chem.* **2023**, *929*, 117124, doi:https://doi.org/10.1016/j.jelechem.2022.117124.
2. Chen, W.; Zhu, M.; Liu, Q.; Guo, Y.; Wang, H.; Wang, K. Fabricating photoelectrochemical aptasensor for sensitive detection of aflatoxin B1 with visible-light-driven BiOBr/nitrogen-doped graphene nanoribbons. *J. Electroanal. Chem.* **2019**, *840*, 67-73, doi:https://doi.org/10.1016/j.jelechem.2019.03.033.
3. Wu, J.; Xie, Z.; Li, M.; Lin, Y.; Tan, X.; Huang, K. Molecularly imprinted photoelectrochemical sensing supported by Bi2S3/Bi2O2CO3 direct Z-scheme heterojunction for aflatoxin B1 detection. *Sens. Actuators B Chem.* **2023**, *378*, 133143, doi:https://doi.org/10.1016/j.snb.2022.133143.
4. Yue, Q.; Li, X.; Fang, J.; Li, M.; Zhang, J.; Zhao, G.; Cao, W.; Wei, Q. Oxygen Free Radical Scavenger Ptpd@Pda as a Dual-Mode Quencher of Electrochemiluminescence Immunosensor for the Detection of Afb1. *Anal. Chem.* **2022**, *94*, 11476–11482, doi:10.1021/acs.analchem.2c00788.
5. Tian, D.; Wang, J.; Zhuang, Q.; Wu, S.; Yu, Y.; Ding, K. An electrochemiluminescence biosensor based on Graphitic carbon nitride luminescence quenching for detection of AFB1. *Food Chem.* **2023**, *404*, 134183, doi:https://doi.org/10.1016/j.foodchem.2022.134183.
6. Zhang, J.; Xia, Y.-K.; Chen, M.; Wu, D.-Z.; Cai, S.-X.; Liu, M.-M.; He, W.-H.; Chen, J.-H. A fluorescent aptasensor

based on DNA-scaffolded silver nanoclusters coupling with Zn(II)-ion signal-enhancement for simultaneous detection of OTA and AFB1. *Sens. Actuators B Chem.* **2016**, *235*, 79-85, doi:https://doi.org/10.1016/j.snb.2016.05.061.

7. Jia, Y.; Zhou, G.; Wang, X.; Zhang, Y.; Li, Z.; Liu, P.; Yu, B.; Zhang, J. A metal-organic framework/aptamer system as a fluorescent biosensor for determination of aflatoxin B1 in food samples. *Talanta* **2020**, *219*, 121342, doi:https://doi.org/10.1016/j.talanta.2020.121342.
8. Lerdsri, J.; Chananchana, W.; Upan, J.; Sridara, T.; Jakmunee, J. Label-free colorimetric aptasensor for rapid detection of aflatoxin B1 by utilizing cationic perylene probe and localized surface plasmon resonance of gold nanoparticles. *Sens. Actuators B Chem.* **2020**, *320*, 128356, doi:https://doi.org/10.1016/j.snb.2020.128356.
